# Sunset Sign Due to Intraventricular Tension Pneumocephalus: A Key Clue to Evaluating Delayed Emergence After General Anesthesia

**DOI:** 10.7759/cureus.74829

**Published:** 2024-11-30

**Authors:** Michael Kaplan, Pratik V Patel, Monica S Vavilala, Abhijit V Lele

**Affiliations:** 1 Anesthesiology and Pain Medicine, Harborview Medical Center, Seattle, USA

**Keywords:** craniotomy, delayed emergence, external ventricular drain (evd), gaze paresis, general anesthesia, parinaud’s syndrome, pineal gland tumor, sunset sign

## Abstract

Prompt emergence from general anesthesia is crucial after neurosurgical procedures, such as craniotomies, to facilitate timely neurological evaluation for identification of intraoperative complications. Delayed emergence can be caused by residual anesthetics, metabolic imbalances, and intracranial pathology, for which an eye examination can provide early diagnostic clues. The sunset sign (or setting sun sign), characterized by a downward deviation of the eyes, can be an early indicator of raised intracranial pressure (ICP) or midbrain compression, as is commonly observed in states of hydrocephalus or periaqueductal or tectal plate dysfunction. A 50-year-old woman with a history of headaches, diplopia, and Parinaud syndrome presented with a pineal mass and underwent an occipital and suboccipital craniotomy with endoscopically-assisted tumor resection. The procedure was managed with neurophysiological monitoring to detect surgical compromise on neurophysiological function. An external ventricular drain (EVD) was placed for cerebrospinal fluid (CSF) drainage to facilitate brain relaxation and operative intervention. Blood loss was estimated to be 200 ml. Thirty minutes after surgery, the patient did not open her eyes to verbal commands despite the cessation of anesthetics significantly earlier. Eye examination revealed an intermittent downward gaze, recognized as the sunset sign.

Arterial blood gas results and metabolic parameters were within normal limits, shifting the focus to possible intracranial complications as the source of her delayed emergence. Consequently, an emergent head computer tomography (CT) was ordered, and the EVD was clamped and not monitored for transport. The CT scan revealed tension pneumocephalus compressing the midbrain. The patient was transferred to the neurocritical care unit, where the admission ICP measured from the EVD was 50 mmHg. Initial critical care treatment included maintaining sedation, CSF drainage via the EVD, 100% oxygen, and head of bed at zero degrees. The patient underwent an MRI brain approximately six hours post-operatively, revealing restricted diffusion in the bilateral medial thalamic regions. The patient was successfully extubated on postoperative day one. Over the following 48 hours, the sunset sign disappeared, the tension pneumocephalus resolved, ICP normalized, and the patient’s neurological status gradually improved.

Delayed emergence after neurosurgical procedures can be multifactorial, and eye movement abnormalities like the sunset sign can offer early diagnostic clues. In this case, the sunset sign occurred from elevated ICP due to tension pneumocephalus, a rare but serious postoperative complication. Early recognition of the sunset sign and immediate neuroimaging allowed for prompt relief of intracranial hypertension, highlighting the importance of incorporating detailed ocular assessments into postoperative evaluations. The sunset sign is an important clinical marker of increased ICP and midbrain dysfunction, warranting urgent investigation. This case underscores the need for early, thorough postoperative assessment, including eye examination, to identify and manage potential complications that may delay emergence from general anesthesia. Eye examination may be warranted as part of routine neurological evaluation during emergence from general anesthesia.

## Introduction

Prompt emergence from general anesthesia is desired after neurosurgical procedures to assess neurological function and identify early perioperative complications. Delayed emergence, where patients do not regain consciousness or normal responsiveness within an expected timeframe, can result from many factors, including residual anesthetics, metabolic imbalances, and/or intracranial pathology. One key clinical feature that can provide early clues in evaluating delayed emergence is the eye examination.

The sunset sign, characterized by a conjugate downward deviation of the eyes, is often an indicator of raised intracranial pressure (ICP) or tectal plate compression. Typically occurring in hydrocephalus or midbrain dysfunction cases, this sign can serve as an essential diagnostic indication during the emergence process, signaling the need for immediate investigation and intervention [1-4]. This report presents a case of delayed emergence after general anesthesia for pineal tumor resection, where the sunset sign was instrumental in identifying an underlying cause of delayed emergence-tension pneumocephalus. Pneumocephalus has been reported in patients undergoing neurosurgical procedures, as well as traumatic and non-traumatic pathologies [5-9]. This case highlights the significance of incorporating thorough pupillary and eye movement assessments in postoperative neurological evaluations to guide timely and appropriate management.

## Case presentation

Patient presentation

**Figure 1:**
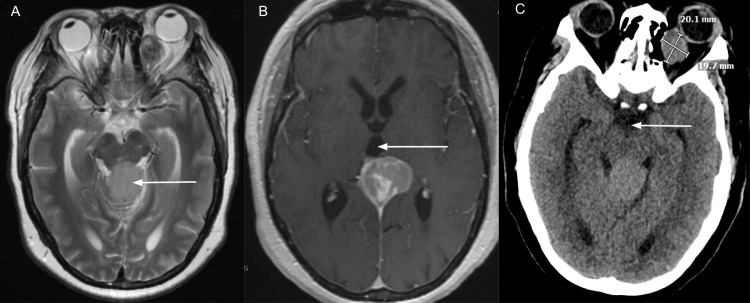
Preoperative MRI and CT images depicting the pineal mass and mass effect on surrounding tissues. A: T2-weighted magnetic resonance imaging (MRI) with arrow depicting pineal gland tumor compressing dorsal midbrain, B: T1-weighted MRI with arrow depicting dilated third ventricle, C: Non-contrast computed tomography of the brain showing cerebrospinal fluid space around the midbrain.

A 50-year-old woman, 5 feet 3 inches tall, weighing 117.9 kilograms, with a history of progressive headaches, diplopia, and vertical gaze palsy, presented for evaluation of a pineal tumor. Neuroimaging confirmed the presence of a 3.3x3.7x4.0 cm pineal mass with a mass effect on the midbrain (Figure 1). Preoperative neurological examination revealed signs of Parinaud syndrome, including upward gaze palsy, but no other significant deficits. Preoperative history was significant for type 2 diabetes mellitus and chronic iron deficiency anemia. 

Anesthetic management

After placing the monitors recommended by the American Society of Anesthesiologists, the patient was preoxygenated with 100% oxygen. An intravenous induction was performed using propofol, fentanyl, and lidocaine. Mask ventilation was performed with an oropharyngeal airway followed by tracheal intubation using a video laryngoscope with a 7.0 cuffed endotracheal tube placement. General anesthesia was maintained using a propofol and remifentanil infusion. The patient was positioned far lateral, and all pressure points were padded. Neurophysiological monitoring consists of motor-evoked potential (MEP), somatosensory evoked potentials (SEP), and electroencephalography (EEG). Two soft bite blocks were placed between the molars, one on each side. The dose of propofol was titrated based on the raw and processed electroencephalography to avoid burst suppression and maintain adequate anesthetic depth. The depth of anesthesia was confirmed by the presence of dominant alpha and delta rhythms in conjunction with the neurophysiologist's interpretation of spontaneous electromyography and responses to MEPs. Remifentanil was dosed at 0.2 mcg/kg/min. Brain relaxation was facilitated using mannitol (0.5 mg/kg bolus). Phenytoin (15 mg/kg bolus) was administered for seizure prophylaxis. The neurosurgeon placed an external ventricular drain. The patient received 4237 ml of crystalloids and was transfused with one unit of packed red blood cells during the procedure. After the conclusion of the neurophysiological monitoring (after dural closure) and considering the context-sensitive half-life of propofol, the general anesthetic technique was changed to inhaled sevoflurane, and propofol remained off for the last hour of the procedure. Analgesia was provided by acetaminophen (1gm intravenously) without the administration of any long-acting opioids.

Emergence from general anesthesia

Thirty minutes after the conclusion of the surgical procedure and one hour after propofol was discontinued, end-tidal sevoflurane concentration was zero, and quantitative train of four surface electromyography showed full reversal from neuromuscular blockade. The patient had swallowing movements, with occasional spontaneous movements in all four extremities, but could not follow commands such as “open your eyes,” performing thumbs up, or holding up two fingers. At the same time, serial examinations of the eyes for pupillary changes and gaze deviations revealed an intermittent downward gaze. Given that the differential for delayed emergence included a metabolic etiology, an arterial blood gas sample was collected, which showed pH 7.38, PCO_2_ 31, PaO_2_ 247, sodium 138mEq/L, and glucose 95mg/dl; the core temperature was 36.8 degrees Celcius. Eliminating the possibility of the effect of residual anesthetic and metabolic abnormalities, we interpreted her eye exam as being positive for the sunset sign. Additionally, the external ventricular drain (EVD) was noted not to be draining cerebrospinal fluid. In consultation with the neurosurgical team, we obtained an urgent computerized tomography of the brain due to concern for increased ICP.

**Figure 2:**
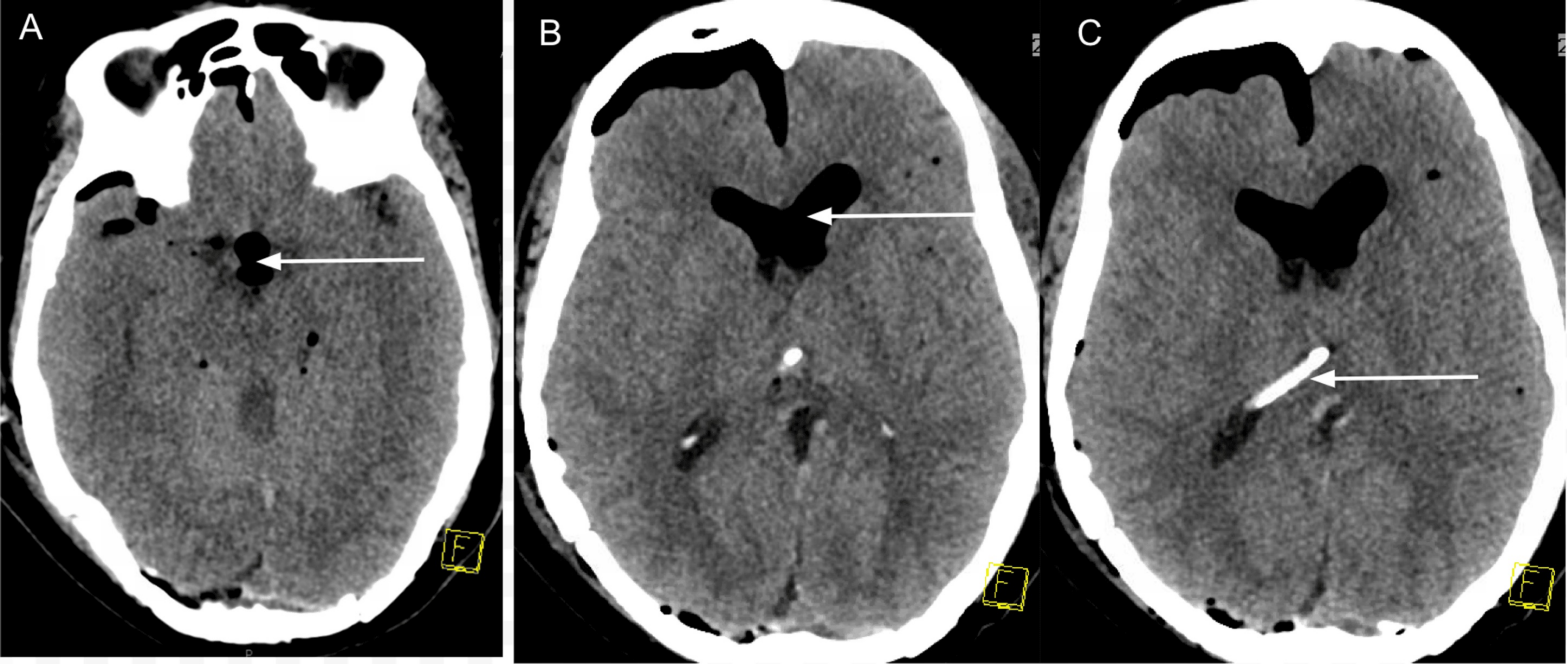
Postoperative computerized non-contrast tomography of the head demonstrating tension pneumocephalus involving the intraventricular space. A: Arrow indicates intraventricular pneumocephalus compressing the ventral midbrain, B: Arrow indicates pneumocephalus in both lateral ventricles and subdural space, C: Arrow indicates proximal end of the external ventricular drain.

As shown in Figure 2, the immediate postoperative CT scan revealed tension pneumocephalus in the intraventricular system, compressing the midbrain and causing the effacement of basal cisterns. The results were unavailable when the patient was transported to the intensive care unit. The mass effect was worse than in the preoperative CT findings. 

**Figure 3:**
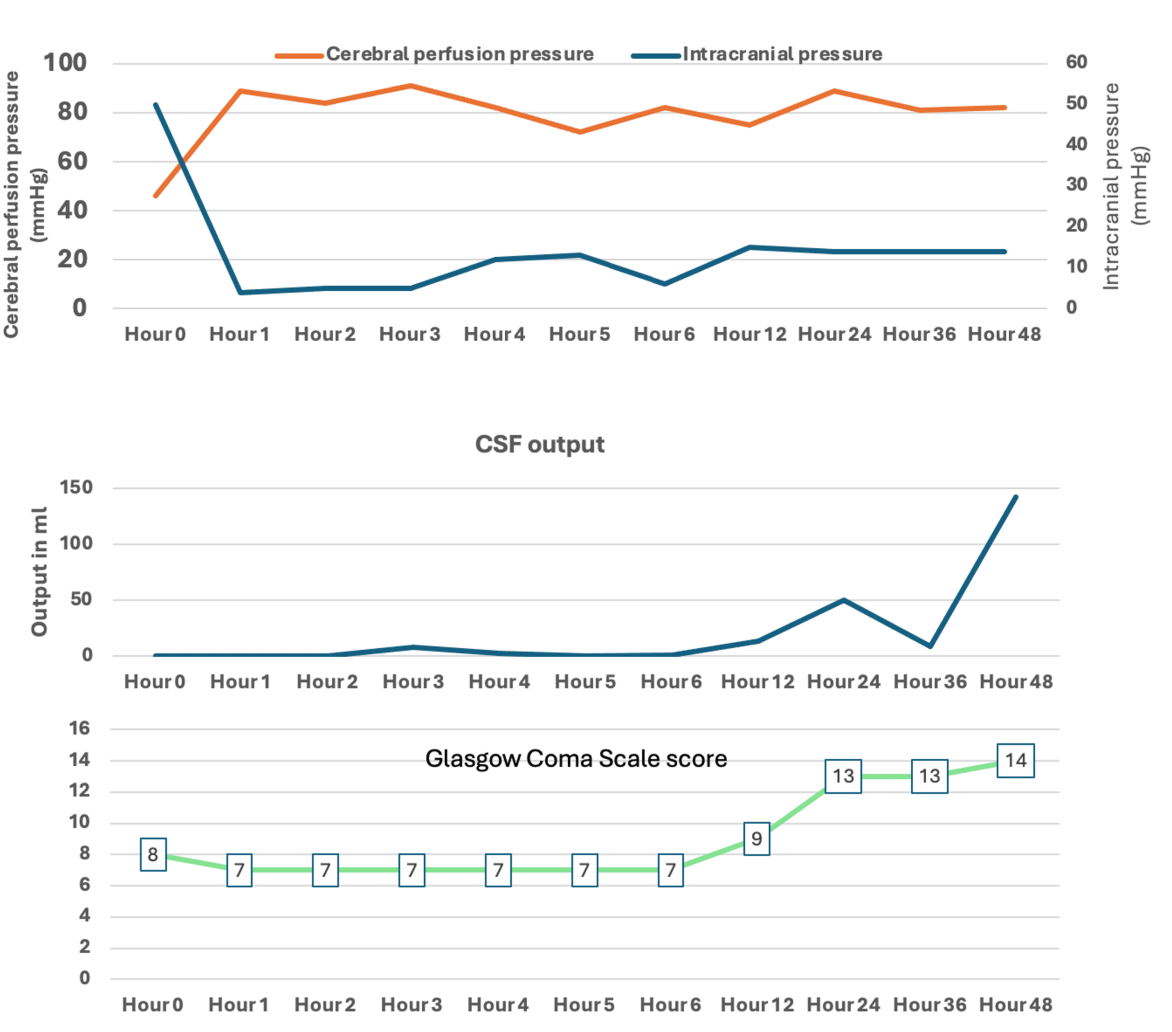
A timeline of postoperative neurological assessments and neurophysiological monitoring data. CSF: cerebrospinal fluid.

After the head CT scan, the patient was transferred to the neurocritical care unit on a propofol infusion, and the first intracranial pressure (ICP) transduced was 50 mmHg. The patient received sedation with propofol, was maintained on 100% inspired oxygen, maintaining PaCO_2_ 35-40 mmHg, and the head of the bed kept at zero degrees to treat the tension pneumocephalus as noted on the immediate postoperative CT scan. Normobaric hyperoxia therapy (with 100% inspired oxygen) was used to denitrogenate the air-lock. In addition, we used the EVD to decompress the intraventricular tension pneumocephalus. As shown in Figure 3, after her initial immediate stabilization upon arrival to the ICU and flushing of the ventriculostomy drainage system by the neurosurgery team, her ICU stay was characterized by a gradual increase in the cerebrospinal fluid (CSF) output with successful ICP and cerebral perfusion pressure (CPP) maintenance. The patient's level of consciousness continued to improve, and she was successfully extubated on the first postoperative day (Figure 3).

The EVD was kept open to continuous drainage and set at +20 cm H_2_0 above the external auditory meatus. The Glasgow Coma Scale (GCS) score, which initially was 7, remained below 8 for several hours post-procedure and then improved by 24 hours, which coincided with the increase in CSF output, implying resolution of the “air-lock” and tension pneumocephalus by 24 hours post-procedure. The improvement in GCS was likely due to several factors, including maintaining ICP within normal limits, maintaining CPP, and an open EVD to allow egress air from the intraventricular space.

**Figure 4:**
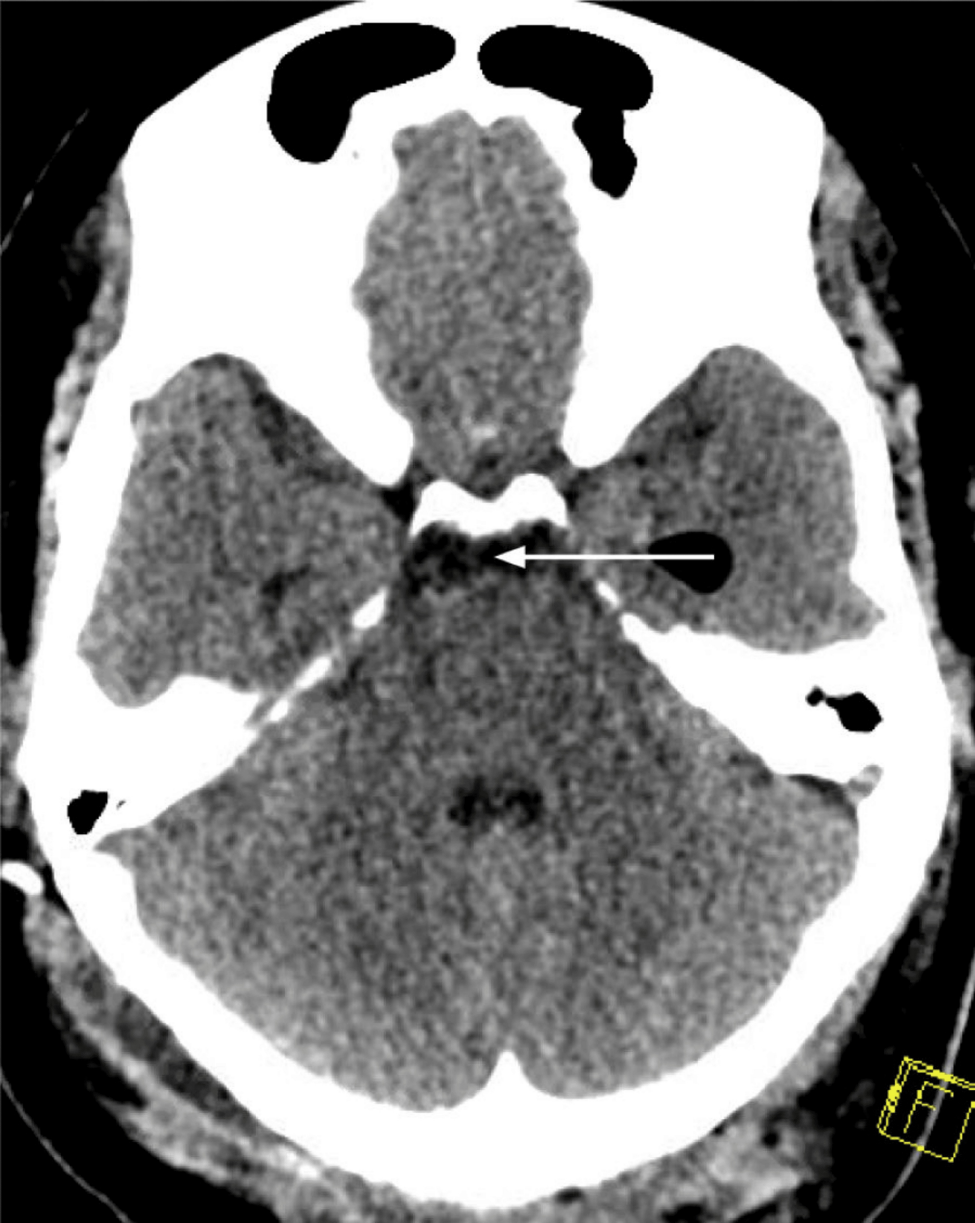
Non-contrast tomography of the head with resorption of intraventricular pneumocephalus. Arrow depicts resorption of air around the midbrain.

As demonstrated in Figure 4, her tension pneumocephalus and the sunset sign completely resolved within 48 hours after the procedure. The subsequent intrahospital stay was notable for the failure of tolerance of the EVD clamp trial and the placement of a ventriculoperitoneal shunt five days after the tumor resection procedure.

## Discussion

Causes of delayed emergence following neurosurgical procedures can be multifactorial, ranging from residual anesthetic effects to surgical complications such as seizures, cerebral edema, intracerebral hemorrhage, cerebral ischemia, or hydrocephalus/pneumocephalus. Prompt identification of high ICP as a cause of delayed emergence allows for early intervention to mitigate neurologic worsening. Thorough neurological examination, including the evaluation of eye gaze, can offer valuable early clues during the emergence process from general anesthesia. This is the first report, to the best of our knowledge, of a postoperative sunset sign used to diagnose tension pneumocephalus in the intraventricular space as the probable cause of delayed emergence from general anesthesia. Our findings also underscore the importance of timely reading of head CT scans and unclamping indwelling EVDs to decrease ICP.

The sunset sign is a hallmark of increased ICP, often associated with tectal compression in the midbrain. The sunset sign results from the inability of the superior rectus and inferior oblique muscles to act synchronously, leading to a downward deviation of the eyes. While the sunset sign is classically diagnostic of chronic hydrocephalus in children, its appearance in adults after neurosurgery reflects intraoperative complications and warrants urgent post-operative investigation and management [1-4]. We postulate that the intraventricular pneumocephalus that developed at some point during neurosurgery created an airlock. As the EVD was closed to CSF drainage at the end of the case (while the ICP was not being monitored), this action may have increased ICP, supported by the fact that the first recorded postoperative ICP in the ICU was 50 mmHg. When the EVD was opened to CSF drainage, and as air in the intraventricular space was able to be resorbed, the ICPs were noted to be reduced to <15 mmHg, which correlated with the resolution of the sunset sign and relieved compression on the arousal structures, ultimately improving the Glasgow Coma Scale score over the next 24-48 hours in the postoperative period.

During the emergence from a general anesthetic, arousal pathways are activated in a caudal-rostral manner, with limb movements observed before swallowing movements, which are observed before dysconjugate eye movements. The eye-gaze transition from dysconjugate to conjugate gaze is often observed during the emergence period, as anesthesiologists frequently use these signs to assess for extubation readiness [10]. We want to point out that the sunset sign is not a normal phenomenon during emergence from the anesthetic, and its presence should alert the anesthesiologist about a critical intracranial pressure elevation affecting the midbrain [11]. Hence, if observed, it is prudent not to ignore it, and it should be promptly reported to the neurosurgical team. It can be hypothesized that without tension pneumocephalus, the patient may have continued to demonstrate Parinaud’s without the sunset sign since her preoperative upward gaze palsy persisted in the postoperative period. The timely recognition of the sunset sign prompted immediate imaging, revealing significant pneumocephalus. In the ICU, the drainage of CSF from EVD facilitated a reduction in the intraventricular pneumocephalus. It took 24 hours for the pneumocephalus to reduce and 48 hours for the GCS to improve from 7 to 13. We include a comparison of the sunset sign with other phenomena and include the relevant clinical signs/symptoms, neuro-anatomy, and clinical relevance in Table 1. 

**Table 1:** Gaze disturbances: symptoms, causes, and anatomical associations.

Feature	Gaze transition	Sunset sign	Parinaud’s syndrome	Cranial nerve III palsy	Bilateral cranial nerve VI palsy
Signs and symptoms	Conjugate and dysconjugate gaze	In upward gaze paresis, eyes appear driven downward, with a visible sclera above the iris.	Upward gaze palsy, convergence-retraction nystagmus, light-near dissociation	Ptosis, mydriasis, eye turned outward and downward	Horizontal gaze limitation, horizontal diplopia with lateral gaze
Causes	Emergence from general anesthesia	Increased intracranial pressure, hydrocephalus, brain tumors	Pineal gland tumors, midbrain lesions	Aneurysms (e.g., posterior communicating artery), trauma, ischemic	Increased intracranial pressure, trauma, tumors
Anatomy involved	Pons and midbrain	Midbrain, cranial nerve III pathways, periaqueductal gray matter	Dorsal midbrain (tectum)	Cranial nerve III, midbrain	Cranial nerve VI), pons

The literature on delayed emergence after pineal tumor resection is primarily anecdotal. Cases like this underscore the need for early recognition of immediate postoperative complications such as hydrocephalus or pneumocephalus. When pneumocephalus creates a mass effect, elevated ICP can compress vital structures, impair arousal pathways, and delay emergence from general anesthesia. Timely recognition of this complication through clinical signs, such as the sunset sign, can facilitate diagnosis and management to prevent neurological worsening by allowing for swift decompression and ICP management. This case also highlights the importance of eye examinations in patients emerging from general anesthesia, especially those in whom the emergence is delayed. An eye examination should be part of the comprehensive neurological assessment and workup for other causes of delayed emergence [12]. The presence of a sunset sign should result in the measurement of ICP if an EVD is present.

## Conclusions

This case report highlights the importance of recognizing the sunset sign as a critical early indicator of increased ICP following pineal and other midbrain tumor resections and as a cause of delayed emergence from general anesthesia due to tension pneumocephalus. Delayed emergence from general anesthesia requires careful clinical evaluation, and ocular signs such as the sunset sign can provide valuable clues to underlying neurological disturbances. Timely neuroimaging and intervention with CSF drainage via a functioning EVD should be considered in the presence of the sunset sign to diagnose intracranial hypertension emergently and to treat intraventricular tension pneumocephalus. This case is a reminder that the sunset sign should prompt the measurement of ICP throughout the continuum of care, including during transport. If an EVD is present, timely relief of intracranial hypertension may prevent an ICP crisis.
